# Supplementary material for: Real-space chemistry on quantum computers: a fault-tolerant algorithm with adaptive grids and a transcorrelated extension
Source: Digit Discov. 2026 Sep 17;5(11):5097–116. doi: 10.1039/d6dd00002a (PMC13651879; doi:10.1039/d6dd00002a)
Supplement: Supplementary file 4 [file DD-005-D6DD00002A-s002.pdf]

Supplementary Information (SI) for Digital Discovery.
This journal is © The Royal Society of Chemistry 2026

Supplementary Information for

Real-Space Chemistry on Quantum Computers: A Fault-Tolerant Algorithm with Adaptive Grids and Transcorrelated Extension

Appendix A: Block-Encoding of the Operators

1. Standard Molecular Hamiltonian

The block-encoding of the molecular Hamiltonian can be constructed using the systematic Pauli LCU decomposition in the first quantization, as introduced in [1, 2], and is valid for arbitrary basis sets, including one that diagonalizes the potential energy. We quote the expression in [2] for the Pauli LCU decomposition of a Hamiltonian that can be decomposed into a one-body term and a diagonal two-body term:

$$\bar{\mathbf{H}} = \sum_{m,n=0}^{N-1} \omega_{mn} \sum_{i=0}^{\eta-1} \prod_{b=0}^{\log N-1} \mathbf{X}_{i\log N+b}^{m_b} \mathbf{Z}_{i\log N+b}^{n_b} + \frac{1}{2} \sum_{m,p=0}^{N-1} \gamma_{mp} \sum_{i\neq j}^{\eta-1} \prod_{b=0}^{\log N-1} \mathbf{Z}_{i\log N+b}^{m_b} \mathbf{Z}_{j\log N+b}^{p_b} \tag{A1}$$

where m_b is the b-th bit in the binary representation of m, likewise for n_b and p_b, and $\mathbf{X}_i$ and $\mathbf{Z}_i$ are Pauli operators acting on the i-th qubit:

$$\mathbf{X}_i = \mathbf{I}_{2^i} \otimes \mathbf{X} \otimes \mathbf{I}_{2^{\eta\log N-i-1}}, \qquad \mathbf{Z}_i = \mathbf{I}_{2^i} \otimes \mathbf{Z} \otimes \mathbf{I}_{2^{\eta\log N-i-1}}. \tag{A2}$$

The explicit expressions of the coefficients are

$$\omega_{mn} = \frac{1}{N} \sum_{x=0}^{N-1} (-1)^{x\odot n} \bar{\mathbf{T}}_{m\oplus x,x}, \qquad \gamma_{mp} = \frac{1}{N^2} \sum_{x,y=0}^{N-1} (-1)^{(m\odot x)+(y\odot p)} \mathbf{W}_{xy} \tag{A3}$$

In Eqs. (A3), $\oplus$ is the bitwise XOR operation and $\odot$ is the bitwise dot product. The expressions of $\bar{\mathbf{T}}_{mn}$ and $\mathbf{W}_{mp}$ are given by equations (21), (16) and (17). One can reduce the number of coefficients to load by neglecting terms with identity Pauli strings, since they only contribute to a constant shift of the eigenvalue. This is done by not considering coefficients for which both indices are zero. Also, one can incorporate repeating strings in the one-body and two-body terms into a single coefficient, which corresponds to that of the string containing a $\mathbf{Z}$ gate on only one of the qubits. Noticing that $\gamma_{mp} = \gamma_{pm}$, we can rewrite the LCU decomposition of the Hamiltonian with these modifications:

$$\bar{\mathbf{H}}_{LCU} = \sum_{m,n=0}^{N-1} \omega'_{mn} \sum_{i=0}^{\eta-1} \prod_{b=0}^{\log N-1} \mathbf{X}_{i\log N+b}^{m_b} \mathbf{Z}_{i\log N+b}^{n_b} + \frac{1}{2} \sum_{m,p=1}^{N-1} \gamma'_{mp} \sum_{i\neq j}^{\eta-1} \prod_{b=0}^{\log N-1} \mathbf{Z}_{i\log N+b}^{m_b} \mathbf{Z}_{j\log N+b}^{p_b} \tag{A4}$$

where

$$\omega'_{mn} = \begin{cases} 0 & \text{if } m=n=0, \\ \omega_{0n} + (N-1)\gamma_{0n} & \text{if } m=0,\ n\neq 0, \\ \omega_{mn} & \text{otherwise,} \end{cases} \qquad \gamma'_{mp} = \begin{cases} 0 & \text{if } m=0 \text{ or } p=0, \\ \gamma_{mp} & \text{otherwise.} \end{cases} \tag{A5}$$

The subscript LCU is added to differentiate between the true Hamiltonian and the one without identity strings.

2. Transcorrelated Molecular Hamiltonian

We adopt a similar approach to block encode the transcorrelated Hamiltonian using an adapted LCU decomposition:

$$\begin{aligned}\tilde{\mathbf{H}} = {} & \sum_{m,n=0}^{N-1} \tilde{\omega}_{mn} \sum_{i=0}^{\eta-1} \prod_{b=0}^{\log N-1} \mathbf{X}_{i\log N+b}^{m_b} \mathbf{Z}_{i\log N+b}^{n_b} + \frac{1}{2} \sum_{m,n,p,q=0}^{N-1} \tilde{\gamma}_{mnpq} \sum_{i\neq j}^{\eta-1} \prod_{b=0}^{\log N-1} \mathbf{X}_{i\log N+b}^{m_b} \mathbf{Z}_{i\log N+b}^{n_b} \mathbf{X}_{j\log N+b}^{p_b} \mathbf{Z}_{j\log N+b}^{q_b} \\ & - \frac{1}{3} \sum_{m,p,t=0}^{N-1} \tilde{\zeta}_{mpt} \sum_{i\neq j\neq k}^{\eta-1} \prod_{b=0}^{\log N-1} \mathbf{Z}_{i\log N+b}^{m_b} \mathbf{Z}_{j\log N+b}^{p_b} \mathbf{Z}_{k\log N+b}^{t_b}\end{aligned} \tag{A6}$$

The explicit expressions for the coefficients are given by:

$$\tilde{\omega}_{mn} = \frac{1}{N}\sum_{x=0}^{N-1}(-1)^{x\odot n}\tilde{\mathbf{T}}_{m\oplus x,x} \tag{A7}$$

$$\tilde{\gamma}_{mnpq} = \frac{1}{N^2}\sum_{x,y=0}^{N-1}(-1)^{(x\odot n)+(y\odot q)}\tilde{\mathbf{W}}_{m\oplus x,x,p\oplus y,y} \tag{A8}$$

$$\tilde{\zeta}_{mpt} = \frac{1}{N^3}\sum_{x,y,z=0}^{N-1}(-1)^{(x\odot m)+(y\odot p)+(z\odot t)}\tilde{\mathbf{B}}_{mpt} \tag{A9}$$

$$\begin{aligned}\tilde{\mathbf{H}}_{LCU} = &\sum_{m,n=0}^{N-1}\tilde{\omega}'_{mn}\sum_{i=0}^{\eta-1}\prod_{b=0}^{\log N-1}\mathbf{X}^{m_b}_{i\log N+b}\mathbf{Z}^{n_b}_{i\log N+b} + \frac{1}{2}\sum_{m,n,p,q=0}^{N-1}\tilde{\gamma}'_{mnpq}\sum_{i\neq j}^{\eta-1}\prod_{b=0}^{\log N-1}\mathbf{X}^{m_b}_{i\log N+b}\mathbf{Z}^{n_b}_{i\log N+b}\mathbf{X}^{p_b}_{j\log N+b}\mathbf{Z}^{q_b}_{j\log N+b}\\ &-\frac{1}{3}\sum_{m,p,t=1}^{N-1}\tilde{\zeta}'_{mpt}\sum_{i\neq j\neq k}^{\eta-1}\prod_{b=0}^{\log N-1}\mathbf{Z}^{m_b}_{i\log N+b}\mathbf{Z}^{p_b}_{j\log N+b}\mathbf{Z}^{t_b}_{k\log N+b}\end{aligned} \tag{A10}$$

where

$$\tilde{\omega}'_{mn} = \begin{cases}0 & \text{if } m=n=0\\ \tilde{\omega}_{0n} + \frac{1}{2}(N-1)(\tilde{\gamma}_{0n00}+\tilde{\gamma}_{000n}) - \frac{1}{3}(N-1)^2(\tilde{\zeta}_{n00}+2\tilde{\zeta}_{00n}) & \text{if } m=0\\ \tilde{\omega}_{mn} + \frac{1}{2}(N-1)(\tilde{\gamma}_{mn00}+\tilde{\gamma}_{00mn}) & \text{otherwise}\end{cases} \tag{A11}$$

$$\tilde{\gamma}'_{mnpq} = \begin{cases}0 & \text{if } m=n=0 \text{ or } p=q=0\\ \tilde{\gamma}_{0n0q} - \frac{1}{3}(N-1)(\tilde{\zeta}_{0nq}+2\tilde{\zeta}_{nq0}) & \text{if } m=p=0\\ \tilde{\gamma}_{mnpq} & \text{otherwise}\end{cases} \tag{A12}$$

$$\tilde{\zeta}'_{mpt} = \begin{cases}0 & \text{if } m=0 \text{ or } p=0 \text{ or } t=0\\ \tilde{\zeta}_{mpt} & \text{otherwise}\end{cases} \tag{A13}$$

Similarly to the two-body LCU expansion derived in [2], one can straightforwardly write one for the three-body term. Note that $\tilde{\mathbf{T}}_{mn}$ and $\tilde{\mathbf{W}}_{mnpq}$ contain non-Hermitian terms, and the latter two-body matrix is no longer diagonal. As before, we remove identity Pauli strings and repeating terms to get fewer coefficients to load. For the three-body term, the symmetry $\tilde{\zeta}_{mpt} = \tilde{\zeta}_{mtp}$ can be exploited to obtain Eqs. (A10)–(A13).

Appendix B: Expression of Differential Operators in the Voronoi Finite Volume Scheme

Consider the molecular Schrödinger equation in (1) and (2):

$$\sum_{i=0}^{\eta-1}\Big(-\frac{1}{2}\nabla^2_{\mathbf{x}_i}\Psi(\ldots,\mathbf{x}_i,\ldots) - \sum_{\alpha=0}^{M-1}\frac{\mathcal{Z}_\alpha}{|\mathbf{x}_i-\mathbf{R}_\alpha|}\Psi(\ldots,\mathbf{x}_i,\ldots) + \sum_{j>i}^{\eta-1} = E\Psi(\ldots,\mathbf{x}_i,\ldots) \tag{B1}$$

and suppose the entire space of interest is filled with arbitrarily distributed points $\{\mathbf{r}_m\}_{m=0}^{N-1}$, forming a Voronoi diagram in $\mathbb{R}^3$. We take the volume integral over the Voronoi cells $\{\mathrm{Vor}(\mathbf{r}_{m_i})\}_{i=0}^{\eta-1}$ occupied by each electron on both sides:

$$\begin{aligned}
&\sum_{i=0}^{\eta-1}\Big(-\frac{1}{2}\int_{\mathrm{Vor}(\mathbf{r}_{m_0})}\cdots\int_{\mathrm{Vor}(\mathbf{r}_{m_i})}\cdots\int_{\mathrm{Vor}(\mathbf{r}_{m_{\eta-1}})}\nabla^2_{\mathbf{x}_i}\Psi(\mathbf{x}_0,\ldots,\mathbf{x}_i,\ldots,\mathbf{x}_{\eta-1})\,d\mathbf{x}_0\ldots d\mathbf{x}_i\ldots d\mathbf{x}_{\eta-1}\\
&-\sum_{\alpha=0}^{M-1}\int_{\mathrm{Vor}(\mathbf{r}_{m_0})}\cdots\int_{\mathrm{Vor}(\mathbf{r}_{m_i})}\cdots\int_{\mathrm{Vor}(\mathbf{r}_{m_{\eta-1}})}\frac{\mathcal{Z}_\alpha}{|\mathbf{x}_i-\mathbf{R}_\alpha|}\Psi(\mathbf{x}_0,\ldots,\mathbf{x}_i,\ldots,\mathbf{x}_{\eta-1})\,d\mathbf{x}_0\ldots d\mathbf{x}_i\ldots d\mathbf{x}_{\eta-1}\\
&+\sum_{j>i}^{\eta-1}\int_{\mathrm{Vor}(\mathbf{r}_{m_0})}\cdots\int_{\mathrm{Vor}(\mathbf{r}_{m_i})}\cdots\int_{\mathrm{Vor}(\mathbf{r}_{m_{\eta-1}})}\frac{1}{|\mathbf{x}_i-\mathbf{x}_j|}\Psi(\mathbf{x}_0,\ldots,\mathbf{x}_i,\ldots,\mathbf{x}_{\eta-1})\,d\mathbf{x}_0\ldots d\mathbf{x}_i\ldots d\mathbf{x}_{\eta-1}\Big)\\
&=E\int_{\mathrm{Vor}(\mathbf{r}_{m_0})}\cdots\int_{\mathrm{Vor}(\mathbf{r}_{m_i})}\cdots\int_{\mathrm{Vor}(\mathbf{r}_{m_{\eta-1}})}\Psi(\mathbf{x}_0,\ldots,\mathbf{x}_i,\ldots,\mathbf{x}_{\eta-1})\,d\mathbf{x}_0\ldots d\mathbf{x}_i\ldots d\mathbf{x}_{\eta-1}
\end{aligned}\tag{B2}$$

According to the divergence theorem applied to a gradient field, we can express the main integral of the first term inside the sum as

$$\int_{\mathrm{Vor}(\mathbf{r}_{m_i})}\nabla^2_{\mathbf{x}_i}\Psi(\ldots,\mathbf{x}_i,\ldots)\,d\mathbf{x}_i=\int_{\partial\mathrm{Vor}(\mathbf{r}_{m_i})}\nabla_{\mathbf{x}_i}\Psi(\ldots,\mathbf{x}_i,\ldots)\cdot d\mathbf{s}_{m_i}=\sum_{n_i\in\Lambda(m_i)}\int_{\Gamma_{m_in_i}}\nabla_{\mathbf{x}_i}\Psi(\ldots,\mathbf{x}_i,\ldots)\cdot\hat{\mathbf{r}}_{m_in_i}\,d\sigma_{m_in_i}\tag{B3}$$

where $d\mathbf{s}_{m_i}$ is the unit normal area vector of the boundary $\partial\mathrm{Vor}(\mathbf{r}_{m_i})$ of the Voronoi cell $\mathrm{Vor}(\mathbf{r}_{m_i})$. In the last equality, we decomposed this boundary as the union of the Voronoi facets $\Gamma_{m_in_i}$ with all surrounding neighbors to $\mathrm{Vor}(\mathbf{r}_{m_i})$, *ie* $\partial\mathrm{Vor}(\mathbf{r}_{m_i})=\bigcup_{n_i\in\Lambda(m_i)}\Gamma_{m_in_i}$. Note that the unit normal vector $\hat{\mathbf{r}}_{m_in_i}=(\mathbf{r}_{n_i}-\mathbf{r}_{m_i})/|\mathbf{r}_{n_i}-\mathbf{r}_{m_i}|$ is a normal vector of the facet $\Gamma_{m_in_i}$. Now, we divide equation (B2) by the product of the volumes of the Voronoi cells $\{v_{m_i}\}_{i=1}^{\eta}$, and take the limit when each one goes to zero. This consists in evaluating the integrands at the discrete points, multiplied by the integration measure and divided by the product of volumes. For one of the terms in the sum of equation (B3), this gives

$$\lim_{v_{m_i}\to0}\frac{1}{v_{m_i}}\int_{\Gamma_{m_in_i}}\nabla_{\mathbf{x}_i}\Psi(\mathbf{x}_1,\ldots,\mathbf{x}_i,\ldots,\mathbf{x}_\eta)\cdot\hat{\mathbf{r}}_{m_in_i}\,d\sigma_{m_in_i}=\frac{1}{v_{m_i}}\nabla_{\mathbf{r}_{m_i}}\Psi(\mathbf{x}_1,\ldots,\mathbf{x}_{i-1},\mathbf{r}_{m_i},\mathbf{x}_{i+1}\ldots,\mathbf{x}_\eta)\cdot\hat{\mathbf{r}}_{m_in_i}\sigma_{m_in_i}\tag{B4}$$

Proceeding similarly with all other terms, one gets

$$\begin{aligned}
&\sum_{i=0}^{\eta-1}\Big(-\frac{1}{2v_{m_i}}\sum_{n_i\in\Lambda(m_i)}\nabla_{\mathbf{r}_{m_i}}\Psi(\mathbf{r}_{m_0},\ldots,\mathbf{r}_{m_i},\ldots,\mathbf{r}_{m_{\eta-1}})\cdot\hat{\mathbf{r}}_{m_in_i}\,\sigma_{m_in_i}-\sum_{\alpha=0}^{M-1}\frac{\mathcal{Z}_\alpha}{|\mathbf{r}_{m_i}-\mathbf{R}_\alpha|}\Psi(\mathbf{r}_{m_0},\ldots,\mathbf{r}_{m_i},\ldots,\mathbf{r}_{m_{\eta-1}})\\
&+\sum_{j>i}^{\eta-1}\frac{1}{|\mathbf{r}_{m_i}-\mathbf{r}_{m_j}|}\Psi(\mathbf{r}_{m_0},\ldots,\mathbf{r}_{m_i},\ldots,\mathbf{r}_{m_{\eta-1}})\Big)=E\Psi(\mathbf{r}_{m_0},\ldots,\mathbf{r}_{m_i},\ldots,\mathbf{r}_{m_{\eta-1}})
\end{aligned}\tag{B5}$$

The continuous problem in (B1) has thus been reduced to a discretized scheme on the Voronoi diagram. We also approximate the directional derivative with a finite difference, whose error can easily be identified using Taylor's theorem:

$$\nabla_{\mathbf{r}_{m_i}}\Psi(\mathbf{r}_{m_0},\ldots,\mathbf{r}_{m_i},\ldots,\mathbf{r}_{m_{\eta-1}})\cdot\hat{\mathbf{r}}_{m_in_i}=\frac{\Psi(\mathbf{r}_{m_0},\ldots,\mathbf{r}_{n_i},\ldots,\mathbf{r}_{m_{\eta-1}})-\Psi(\mathbf{r}_{m_0},\ldots,\mathbf{r}_{m_i},\ldots,\mathbf{r}_{m_{\eta-1}})}{|\mathbf{r}_{n_i}-\mathbf{r}_{m_i}|}+\mathcal{O}(|\mathbf{r}_{n_i}-\mathbf{r}_{m_i}|)\tag{B6}$$

Combining equations (B5) and (B6), we find the expression of the discretized laplacian operator in (15) and that of the scalar potential energy operators. For the gradient operator, similarly to what done in (B3) and (B4), and using an alternative form of the divergence theorem:

$$\begin{aligned}
&\lim_{v_{m_i}\to0}\frac{1}{v_{m_i}}\int_{\mathrm{Vor}(\mathbf{r}_{m_i})}\nabla_{\mathbf{x}_i}\Psi(\ldots,\mathbf{x}_i,\ldots)\,d\mathbf{x}_i=\lim_{v_{m_i}\to0}\frac{1}{v_{m_i}}\int_{\partial\mathrm{Vor}(\mathbf{r}_{m_i})}\Psi(\ldots,\mathbf{x}_i,\ldots)\big|_{\partial\mathrm{Vor}(\mathbf{r}_{m_i})}\,d\mathbf{s}_{m_i}\\
&=\sum_{n_i\in\Lambda(m_i)}\lim_{v_{m_i}\to0}\frac{1}{v_{m_i}}\int_{\Gamma_{m_in_i}}\Psi(\ldots,\mathbf{x}_i,\ldots)\big|_{\Gamma_{m_in_i}}\hat{\mathbf{r}}_{m_in_i}\,d\sigma_{m_in_i}\\
&=\sum_{n_i\in\Lambda(m_i)}\frac{1}{v_{m_i}}\Psi(\mathbf{r}_{m_1},\ldots,\mathbf{r}_{m_i},\ldots,\mathbf{r}_{m_\eta})\big|_{\Gamma_{m_in_i}}\hat{\mathbf{r}}_{m_in_i}\sigma_{m_in_i}
\end{aligned}\tag{B7}$$

We approximate the value of the wavefunction on the facet $\Gamma_{m_i n_i}$ by the average value

$$\Psi(\mathbf{r}_{m_0}, \ldots, \mathbf{r}_{m_i}, \ldots, \mathbf{r}_{m_{\eta-1}})\big|_{\Gamma_{m_i n_i}} \approx \frac{\Psi(\mathbf{r}_{m_0}, \ldots, \mathbf{r}_{m_i}, \ldots, \mathbf{r}_{m_{\eta-1}}) + \Psi(\mathbf{r}_{m_0}, \ldots, \mathbf{r}_{n_i}, \ldots, \mathbf{r}_{m_{\eta-1}})}{2} \tag{B8}$$

Knowing that $\sum_{n_i} \hat{\mathbf{r}}_{m_i n_i} \sigma_{m_i n_i} = 0$ for any convex polygon, the matrix form of the gradient operator projected on a unit vector $\hat{\mathbf{z}}$, *ie* the matrix representation of the directional derivative along $\hat{\mathbf{z}}$, is given by:

$$\mathbf{D}_{mn}^{(\hat{\mathbf{z}})} = \begin{cases} \dfrac{\sigma_{mn}}{2v_m} \dfrac{\mathbf{r}_m - \mathbf{r}_n}{|\mathbf{r}_m - \mathbf{r}_n|} \cdot \hat{\mathbf{z}} & \text{if } n \in \Lambda(m) \\ 0 & \text{otherwise} \end{cases} \tag{B9}$$

Appendix C: Initial state

In second-quantized encoding, qubits directly represent orbital occupations, making the Hartree–Fock (HF) determinant trivial to prepare. When stronger ground-state support is needed, various techniques exist to load correlated CI states within the second-quantized formalism [3–5]. First-quantized, real-space encoding requires classically computed molecular orbitals to be evaluated over the chosen discretisation, and the corresponding amplitudes loaded to the computational basis of the qubit register. Then, a fermionic antisymmetrization circuit of size $\mathcal{O}(\eta \log \eta \log N)$ and depth $\mathcal{O}(\log \eta \log \log N)$ is applied to the single-electron registers. The key task is efficient loading of Gaussian orbital amplitudes onto the computational basis, a task that can be addressed through careful grid points / Voronoi cell ordering and advanced state loading routines [6–8]. Likewise, CI wavefunctions can be prepared by first loading each Slater determinant with the above procedure and then assembling the CI state using the standard LCU approach with `PREPARE` and `SELECT` oracles.

Appendix D: Further Basis Convergence Simulations

Figure 1 compares the convergence of the hydrogen ground-state energy for three Becke–Lebedev radial exponents $\nu = 1, 2, 3$. Panel (a) shows the non-transcorrelated Hamiltonian, while (b) and (c) use TC Hamiltonians with $\mu_{ne} = 3$ and $\mu_{ne} = 1$, respectively. In each plot the error (in Hartree) is plotted on a logarithmic scale versus the total number of grid points N. The blue band indicates chemical accuracy (≤ 1 mHa) to the FCI-CBS. Introducing the Jastrow factor accelerates convergence, and reducing μ_{ne} ($\mu_{ne} = 1$) achieves the chemical-accuracy threshold several thousand points sooner than the non-TC case. Across all settings the grid with $\nu = 1$ consistently outperforms higher exponents. This suggests that, for this simple system, the radial grid need not be excessively concentrated near the nucleus. This may seem counterintuitive, since larger Becke exponents are commonly used in DFT quadrature. However, the present grid is not an integration rule: it defines the basis on which the differential operator is represented. There is therefore no reason for the exponent that is optimal for DFT quadrature to also be optimal for solving the Hamiltonian ground state problem.

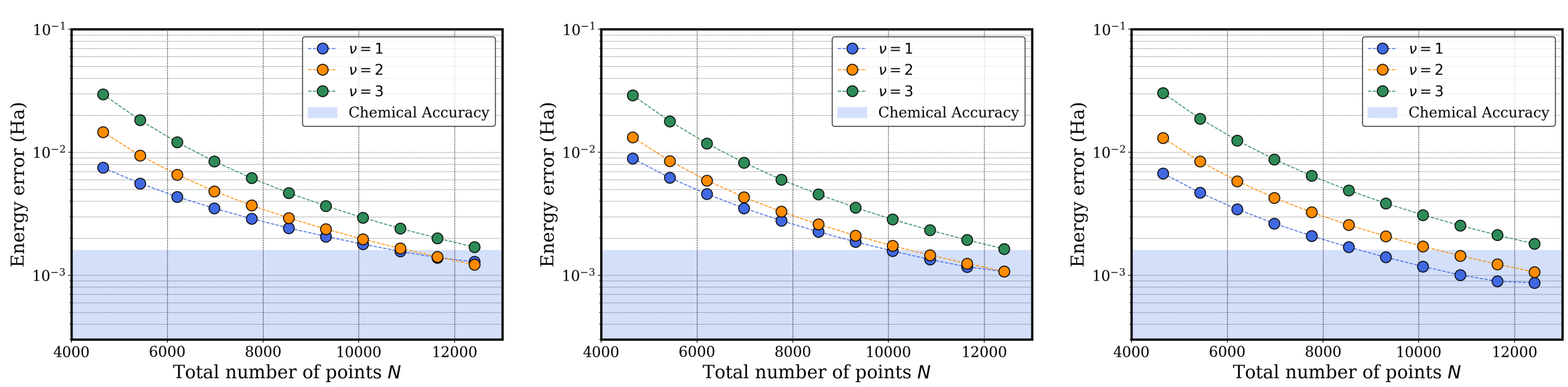

(a) Non-TC (b) $\mu_{ne} = 3$ (c) $\mu_{ne} = 1$

Figure 1: Convergence of the hydrogen ground-state energy for Becke–Lebedev grids with radial exponents $\nu = 1$ (blue), $\nu = 2$ (orange), and $\nu = 3$ (green). The shaded band marks chemical accuracy (1 mHa). Panels compare the non-transcorrelated (a) and transcorrelated Hamiltonians with $\mu_{ne} = 3$ (b) and $\mu_{ne} = 1$ (c).

Figure 2 compares the energy convergence of the transcorrelated helium ground state on Gauss–Legendre and Lebedev radial grids. The Lebedev grid achieves the same level of accuracy several shells earlier than Gauss–Legendre at the same angular resolution, highlighting its better point distribution. As typical in grid-based approaches, the

computed energy approaches the full-CI complete-basis-set limit "from below," with smaller grids underestimating the true energy and each refinement incrementally raising the value toward the FCI-CBS limit. We also report the corresponding non-transcorrelated (NTC) simulations as a baseline. Overall, the TC results show lower errors than their NTC counterparts, confirming that the transcorrelated treatment improves the spatial description of the wave function.

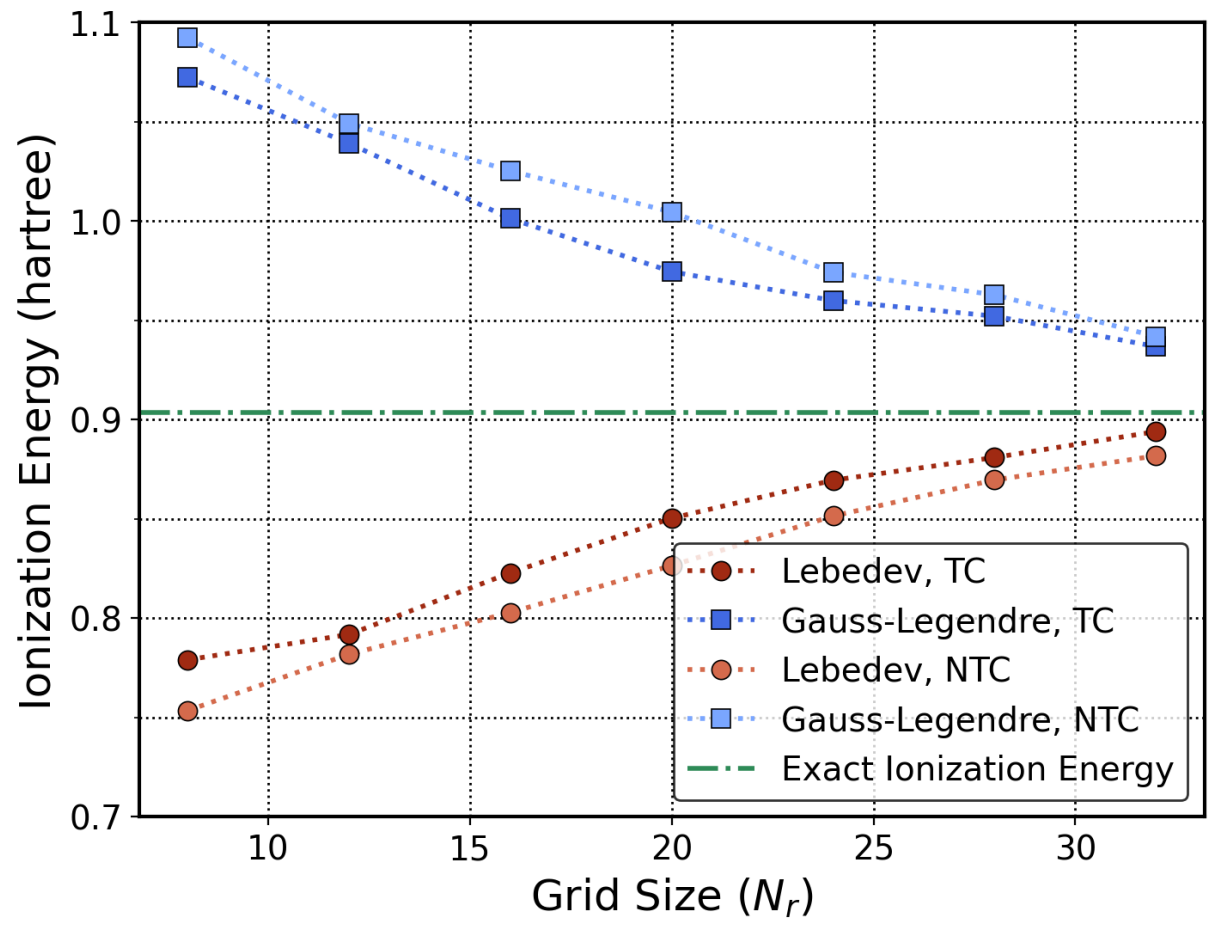

Figure 2: Convergence of the helium first ionization energy with radial grid size for Gauss–Legendre and Lebedev quadratures, comparing transcorrelated (TC) and non-transcorrelated (NTC) simulations (128 angular points each, $\mu_{ee} = 2$, $\mu_{ne} = 1$ for TC). The dash-dotted line marks the complete-basis-set full-CI reference ionization energy of He.

Appendix E: Block Encoding Procedure

Definition E.1 (Linear Combination of Unitaries)**.** Given the matrix representation $\mathbf{H} \in \mathbb{C}^{N\times N}$ of the Hamiltonian (or any other operator), which can be decomposed into a linear combination of unitaries (LCU), *ie* for $L \leq N^2$ and

$$\mathbf{H} = \sum_{l=0}^{L-1} a_l \mathbf{U}_l \tag{E1}$$

where a_l are some complex coefficients and $\mathbf{U}_l$ are $N \times N$ unitary operators, we define the one-norm of the LCU of $\mathbf{H}$ as

$$\lambda = \sum_{l=0}^{L-1} |a_l| \tag{E2}$$

Definition E.2 (Block Encoding of Square Matrices)**.** We refer to $\mathbf{U}_H$ as a $(\lambda, \kappa, \epsilon)$-block encoding of $\mathbf{H} \in \mathbb{C}^{N\times N}$ with $\lambda > 0$ and $\epsilon > 0$ if we have

$$\left\| \frac{\mathbf{H}}{\lambda} - \left(\langle 0|^{\otimes\kappa} \otimes \mathbf{I}_N\right) \mathbf{U}_H \left(|0\rangle^{\otimes\kappa} \otimes \mathbf{I}_N\right) \right\|_2 \leq \epsilon \tag{E3}$$

$\mathbf{I}_N$ is the N by N identity matrix. Sandwiching $\mathbf{U}_H \in \mathbb{C}^{2^\kappa N \,\times\, 2^\kappa N}$ between $|0\rangle^{\otimes\kappa} \otimes \mathbf{I}_N$ and its bra counterpart is just a mathematical way of saying that we extract the upper left N by N block of $\mathbf{U}_H$. When the block-encoding is exact, *ie* $\epsilon = 0$, $\mathbf{U}_H$ becomes a (λ, κ) block encoding and it takes the form

$$\mathbf{U}_H = \begin{pmatrix} \mathbf{H}/\lambda & * \\ * & * \end{pmatrix} \tag{E4}$$

where the blocks denoted by '$*$' do not contain useful information but ensure unitarity. A block encoding $\mathbf{U}_H$ of $\mathbf{H}$ can be obtained by combining the operators `PREP` (preparation) and `SELECT` operations defined as [9]:

$$\texttt{PREP}\,|0\rangle^{\otimes \log L} = \sum_{l=0}^{L-1} \sqrt{\frac{a_l}{\lambda}}\,|l\rangle\,, \texttt{SELECT} = \sum_{l=0}^{L-1} |l\rangle\langle l| \otimes \mathbf{U}_l \tag{E5}$$

and performing fixed-point oblivious amplification on the $|0\rangle^{\otimes\kappa}$ state [10, 11] after constructing the gate

$$(\texttt{PREP}^\dagger \otimes \mathbf{I}_N) \cdot \texttt{SELECT} \cdot (\texttt{PREP} \otimes \mathbf{I}_N) \tag{E6}$$

Block-encoding then enables the construction of a unitary operator

$$\mathbf{Q} = \mathbf{U}_H \begin{pmatrix} \mathbf{I}_{N\times N} & \mathbf{0}_{N\times(2^\kappa-N)} \\ \mathbf{0}_{(2^\kappa-N)\times N} & -\mathbf{I}_{(2^\kappa-N)\times(2^\kappa-N)} \end{pmatrix} \tag{E7}$$

called the *qubitized walk-operator* [12]. When the block-encoding is exact, $\mathbf{Q}$ has eigenvalues equal to $e^{\pm i\cos^{-1}(E_k/\lambda)}$, where E_k are the eigenvalues of $\mathbf{H}$.

Appendix F: Quantum Chebyshev Phase Estimation

In this section, we summarize the method proposed by Low and Su [13] to estimate the eigenvalues of our non-Hermitian Hamiltonian. We recall that this loss of hermiciticty is due to the additional non-Hermitian differential operators (31) and (32) that arise from the transcorrelation transformation and our inability to perform a symmetrization procedure similar to what was done in section II D. This quantum algorithm is only applicable to square matrices with real eigenvalues, which we conjecture is the case for our Hamiltonian due to numerical experiments with exact diagonalization. The method can be extended to eigenvalues that are contained in an area of the complex plane, but for simplicity we will only consider square matrices with real eigenvalues in the interval $[-1; 1]$.

1. Overview

As the transcorrelated Hamiltonian matrix $\tilde{\mathbf{H}} \in \mathbb{R}^{N^\eta \times N^\eta}$ is non-Hermitian matrix, finding its eigenvalues with a quantum algorithm can no longer be accessed through the usual qubitzed QPE procedure. Hence, one needs to consider a larger class of QEVE algorithms. Given an υ qubit register, a state $|\psi\rangle \in \mathbb{C}^{N^\eta}$ that is prepared close to the ground state of $\tilde{\mathbf{H}}$, and a subnormalization constant $\alpha_{\tilde{\mathbf{H}}}$ of $\tilde{\mathbf{H}}$, previous approaches were based on generating (up to a normalization constant) the state [14, 15]

$$\sum_{\ell=0}^{\upsilon-1} |\ell\rangle\, e^{2\pi i\ell \frac{\tilde{\mathbf{H}}}{\alpha_{\tilde{H}}}}\, |\psi\rangle \tag{F1}$$

and measuring the eigenvalue from the phase that is kick-backed onto the ancilla qubits. However, this algorithm suffers from a suboptimal $\mathcal{O}(\epsilon^{-1}\cdot \mathrm{polylog}(\epsilon^{-1}))$ query complexity dependence on ϵ. To resolve this, [13] introduced an alternative QEVE algorithm based on generating *Chebyshev history states*, and whose query complexity regains the $\mathcal{O}(1/\epsilon)$ of QPE. We summarize this *Quantum Chebyshev Phase Estimation* (QCPE) algorithm above and we state the main theorem here.

Theorem F.1 (Quantum Chebyshev Phase Estimation [13])**.** *Let $\mathbf{H}$ be a square matrix with only real eigenvalues such that $\mathbf{H}/\alpha_H$ is a block encoded by O_H with some normalization factor $\alpha_H \geq 2||\mathbf{H}||_2$. Suppose that oracle $O_\psi|0\rangle = |\psi\rangle$ prepares an initial state within distance $\big|\big|\,|\psi\rangle - |\psi_0\rangle\,\big|\big| = \mathcal{O}(\frac{\epsilon}{\alpha_H\alpha_U})$ from an eigenstate $|\psi_0\rangle$ such that $\mathbf{H}|\psi_0\rangle = E_0|\psi_0\rangle$, where*

$$\alpha_U \geq \max_{\ell=0,1\ldots\upsilon-1} ||U_\ell(\frac{\mathbf{H}}{\alpha_H})|| \tag{F2}$$

is an upper bound on the Chebyshev polynomials of the second kind $U_j(x)$ (F8) with $\upsilon = \mathcal{O}(\frac{\alpha_H}{\epsilon})$. Then, E_0 can be estimated with accuracy ϵ and probability $1-p_f$ using

$$\mathcal{O}\Big(\frac{\alpha_H}{\epsilon}\alpha_U \log\big(\frac{1}{p_f}\big)\Big) \tag{F3}$$

queries to controlled-O_H, controlled-O_ψ and their inverses.

2. Preliminaries on Chebyshev Polynomials

Definition F.1 (Chebyshev Polynomials of the First and Second Kinds [16])**.**

- The ℓth Chebyshev polynomial of the first kind $T_j : [-1; 1] \to [-1; 1]$ is defined as:

$$T_\ell(x) = \cos\big(\ell \arccos(x)\big) \tag{F4}$$

with generating function

$$\sum_{\ell=0}^{\infty} T_\ell(x) y^\ell = \frac{1-yx}{1+y^2-2yx} \tag{F5}$$

It is also convenient to define the rescaled Chebyshev polynomials of the first kind:

$$\tilde{T}_\ell(x) = \begin{cases} \frac{1}{2}T_0(x) & \text{if } \ell = 0 \\ T_\ell(x) & \text{if } \ell \geq 1 \end{cases} \tag{F6}$$

with generating function

$$\sum_{\ell=0}^{\infty} \tilde{T}_\ell(x) y^\ell = \frac{1-yx}{2(1+y^2-2yx)} \tag{F7}$$

- The ℓth Chebyshev polynomial of the second kind $U_\ell : [-1;1] \to [-1;1]$ is defined as:

$$U_\ell(x) = \frac{\sin\big((\ell+1)\arccos(x)\big)}{\sin\big(\arccos(x)\big)} \tag{F8}$$

with generating function

$$\sum_{\ell=0}^{\infty} U_\ell(x) y^\ell = \frac{1}{1+y^2-2yx} \tag{F9}$$

3. The Algorithm

Definition F.2 (Chebyshev History State)**.** Let $\mathbf{H}$ be a square matrix with real eigenvalues and subnormalization constant $\alpha_H > \|\mathbf{H}\|_2$. Consider an υ qubit register and an arbitrary state $|\psi\rangle$. The total system is said to form a Chebyshev history state of $\mathbf{H}$ if it can be written as

$$\sum_{\ell=0}^{\upsilon-1} |\ell\rangle \otimes T_\ell\big(\frac{\mathbf{H}}{\alpha_H}\big) |\psi\rangle \tag{F10}$$

up to a normalization constant, where T_ℓ is the ℓth Chebyshev polynomial of the first kind as defined in (F4).

The reason for the name *history state* is the fact that the υ qubits register $|\ell\rangle$ serves as a counter that indicates the order of the Chebyshev polynomial of $\mathbf{H}/\alpha_H$ that is applied on the state $|\psi\rangle$ in the sum. Suppose now that $|\psi\rangle$ is the eigenvector of $\mathbf{H}$ corresponding to its ground state, *ie* $|\psi\rangle = |\psi_0\rangle$ such that $\mathbf{H}|\psi_0\rangle = E_0|\psi_0\rangle$. Then, the history state takes the form

$$\sum_{\ell=0}^{\upsilon-1} |\ell\rangle \otimes T_\ell\big(\frac{\mathbf{H}}{\alpha_H}\big) |\psi_0\rangle = \sum_{\ell=0}^{\upsilon-1} T_\ell\big(\frac{E_0}{\alpha_H}\big) |\ell\rangle \otimes |\psi_0\rangle = \sum_{\ell=0}^{\upsilon-1} \cos(2\pi\ell\phi) |\ell\rangle \otimes |\psi_0\rangle \tag{F11}$$

where $\phi = \frac{1}{2\pi}\arccos\frac{E_0}{\alpha_H}$. We now neglect the $|\psi_0\rangle$ register, whose purpose was to phase-kickback the cosine term to the υ qubit register, and apply a quantum Fourier transform:

$$QFT \sum_{\ell=0}^{\upsilon-1} \cos(2\pi\ell\phi) |\ell\rangle = \frac{1}{2\sqrt{\upsilon}} \sum_{\ell=0}^{\upsilon-1}\sum_{\ell'=0}^{\upsilon-1} \Big(e^{2\pi i\ell'(\phi-\frac{\ell}{\upsilon})} + e^{-2\pi i\ell'(\phi+\frac{\ell}{\upsilon})}\Big) |\ell\rangle \tag{F12}$$

If we scale $\alpha_H > 2||\mathbf{H}||_2$, the authors show that, by measuring ℓ in the computational basis, we obtain an approximate of $\upsilon\phi$ with a success probability greater than 1/2. More precisely, starting from a Chebyshev history state (F16), performing a QFT, and measuring in the computational basis gives a value ℓ satisfying

$$|cmod_1(\frac{\ell}{\upsilon} \pm \phi)| < \frac{\upsilon'}{\upsilon} \tag{F13}$$

with probability at least 0.566 for $\upsilon' \geq 5$. Here, *cmod* refers to the centered modulus function defined as

$$cmod_q(x) = x - q\lfloor \frac{x + \frac{q}{2}}{q} \rfloor \tag{F14}$$

The success probability can then be boosted to at least $1-p_f$ by repeating the procedure $\mathcal{O}(\log(1/p_f))$ times. However, one must first be able to generate Chebyshev history states.

Lemma F.2. *Let* $\mathbf{H}$ *be a square matrix with real eigenvalues and subnormalization constant* $\alpha_H > ||\mathbf{H}||_2$ *and let* $\mathbf{L}_\upsilon = \sum_{\ell=0}^{\upsilon-2} |\ell+1\rangle\langle\ell|$ *be the* $\upsilon \times \upsilon$ *lower shift operator. The matrix version of the operator of the generating function for rescaled Chebyshev polynomials* (F7)

$$G(\frac{\mathbf{H}}{\alpha_H}) = \sum_{\ell=0}^{\upsilon-1} \mathbf{L}_\upsilon^\ell \otimes \tilde{T}_\ell(\frac{\mathbf{H}}{\alpha_H}) \tag{F15}$$

applied on the $|0\rangle \otimes |\psi\rangle$ *state gives the rescaled Chebyshev history state*

$$\sum_{\ell=1}^{\upsilon-1} |\ell\rangle \otimes \tilde{T}_\ell(\frac{\mathbf{H}}{\alpha_H}) |\psi\rangle \tag{F16}$$

Proof. It is a straightforward application of the operator G, noticing that elevating the $\upsilon \times \upsilon$ lower shift operator to the ℓth power gives

$$\mathbf{L}_\upsilon^\ell = \sum_{\ell'=0}^{\upsilon-1-\ell} |\ell+\ell'\rangle\langle\ell'| \tag{F17}$$

□

In order to use the Chebyshev quantum phase estimation algorithm, we thus have to apply this operator to the zeroed υ qubit register. To implement it as a gate on a quantum computer, we first define a padding of the matrix $\mathbf{H}/\alpha_H$:

$$\begin{aligned} Pad(\frac{\mathbf{H}}{\alpha_H}) &= \mathbf{I}_\upsilon \otimes \mathbf{I} + \mathbf{L}_\upsilon^2 \otimes \mathbf{I} - 2\mathbf{L}_\upsilon \otimes \frac{\mathbf{H}}{\alpha_H} \\ &= \begin{pmatrix} \mathbf{I} & 0 & \cdots & \cdots & \cdots & 0 \\ -2\mathbf{H}/\alpha_H & \mathbf{I} & \ddots & \ddots & \ddots & \vdots \\ \mathbf{I} & -2\mathbf{H}/\alpha_H & \mathbf{I} & \ddots & \ddots & \vdots \\ 0 & \mathbf{I} & -2\mathbf{H}/\alpha_H & \mathbf{I} & \ddots & \vdots \\ \vdots & \ddots & \ddots & \ddots & \ddots & \vdots \\ 0 & \cdots & 0 & \mathbf{I} & -2\mathbf{H}/\alpha_H & \mathbf{I} \end{pmatrix} \end{aligned} \tag{F18}$$

and use the following lemma.

Lemma F.3.

$$G(\frac{\mathbf{H}}{\alpha_H}) = Pad(\frac{\mathbf{H}}{\alpha_H})^{-1}.\Big(\frac{\mathbf{I}_\upsilon - \mathbf{L}_\upsilon^2}{2} \otimes \mathbf{I}\Big) \tag{F19}$$

Proof. Inverting the right-hand side of (F18) and multiplying by $\frac{\mathbf{I}_\upsilon - \mathbf{L}_\upsilon^2}{2} \otimes \mathbf{I}$, we obtain the form of the generating function for the rescaled Chebyshev polynomials of (F7) with $x = \mathbf{I}_\upsilon \otimes \frac{\mathbf{H}}{\alpha_H}$ and $y = \mathbf{L}_\upsilon \otimes \mathbf{I}$. □

Since finding the inverse of a matrix, let alone on a quantum computer, is a tedious task, we will get our Chebyshev history state by solving the linear system

$$Pad(\frac{\mathbf{H}}{\alpha_H}) |\Phi\rangle \otimes |\psi\rangle = \frac{|0\rangle - |2\rangle}{2} \otimes |\psi\rangle \tag{F20}$$

Algorithm: Quantum Chebyshev Phase Estimation

1. Construct a block encoding of the target matrix $\mathbf{H}$ and the lower-shift matrix $\mathbf{L}_\upsilon$.
2. Use $\mathbf{L}_\upsilon$ and $\mathbf{H}$ to construct the $\mathrm{Pad}(\mathbf{H}/\alpha_H)$ gate according to Eq. (F18).
3. Prepare the υ-qubit register in the state
$$\frac{|0\rangle - |2\rangle}{\sqrt{2}}.$$
4. Invoke the quantum linear-system solver to solve Eq. (F20) and prepare $|\Phi\rangle$.
5. Perform the quantum Fourier transform and measure in the computational basis.
6. Boost the success probability using median amplification.

Figure 3: Quantum Chebyshev Phase Estimation algorithm.

for a state $|\Phi\rangle \in \mathcal{H}^{2^\upsilon}$. The right-hand side of the equation results from applying the operator $\frac{\mathbf{I}_\upsilon - \mathbf{L}_\upsilon^2}{2} \otimes \mathbf{I}$ to the $|0\rangle \otimes |\psi\rangle$ state. Many quantum linear system solvers have been proposed, but the authors propose to use the one described in [17]. The task of block-encoding the gate $Pad(\mathbf{H}/\alpha_H)$ and the lower shift matrix is one that will not be discussed in this work, as it has been extensively elaborated on in [13].

The general theorem for the Chebyshev phase estimation F.1 takes into account (i) the error from the quantum linear system solver, (ii) the distance between the prepared state $|\psi\rangle$ and the true ground state eigenstate $|\psi_0\rangle$ and (iii) the fact that we are approximating a rescaled Chebyshev history state (F.2) to a normal one (F16). Finally, for preparing an initial state that is close to the true ground state, the authors introduce an algorithm for preparing the ground state of a non-Hermitian matrix using *Quantum EigenValue Transformation* (QEVT). It mainly consists of forming a more general Chebyshev state than the one in (F16), which requires the construction of an extended $Pad(A)$ matrix.

[1] Timothy N Georges, Bjorn K Berntson, Christoph Sünderhauf, and Aleksei V Ivanov. Pauli decomposition via the fast walsh-hadamard transform. *New Journal of Physics*, 27(3):033004, February 2025.

[2] Timothy N Georges, Marius Bothe, Christoph Sünderhauf, Bjorn K Berntson, Róbert Izsák, and Aleksei V Ivanov. Quantum simulations of chemistry in first quantization with any basis set. *npj Quantum Information*, 11(1):55, 2025.

[3] César Feniou, Olivier Adjoua, Baptiste Claudon, Julien Zylberman, Emmanuel Giner, and Jean-Philip Piquemal. Sparse quantum state preparation for strongly correlated systems. *The Journal of Physical Chemistry Letters*, 15(11):3197–3205, 2024.

[4] Norm M. Tubman, Carlos Mejuto-Zaera, Jeffrey M. Epstein, Diptarka Hait, Daniel S. Levine, William Huggins, Zhang Jiang, Jarrod R. McClean, Ryan Babbush, Martin Head-Gordon, and K. Birgitta Whaley. Postponing the orthogonality catastrophe: efficient state preparation for electronic structure simulations on quantum devices. *arXiv preprint arXiv:1809.05523*, 2018.

[5] Stepan Fomichev, Kasra Hejazi, Modjtaba Shokrian Zini, Matthew Kiser, Joana Fraxanet Morales, Pablo Antonio Moreno Casares, Alain Delgado, Joonsuk Huh, Arne-Christian Voigt, Jonathan E. Mueller, and Juan Miguel Arrazola. Initial state preparation for quantum chemistry on quantum computers. *arXiv preprint arXiv:2310.18410*, 2023.

[6] Danial Motlagh and Nathan Wiebe. Generalized quantum signal processing. *PRX Quantum*, 5(2):020368, 2024.

[7] Julien Zylberman and Fabrice Debbasch. Efficient quantum state preparation with walsh series. *Physical Review A*, 109(4):042401, 2024.

[8] Adam Holmes and Anne Y Matsuura. Efficient quantum circuits for accurate state preparation of smooth, differentiable functions. In *2020 IEEE international conference on quantum computing and engineering (QCE)*, pages 169–179. IEEE, 2020.

[9] Guang Hao Low and Isaac L Chuang. Hamiltonian simulation by qubitization. *Quantum*, 3:163, 2019.

[10] Dominic W. Berry, Mária Kieferová, Artur Scherer, Yuval R. Sanders, Guang Hao Low, Nathan Wiebe, Craig Gidney, and Ryan Babbush. Improved techniques for preparing eigenstates of fermionic hamiltonians. *npj Quantum Information*, 4(1), May 2018.

[11] Bao Yan, Shijie Wei, Haocong Jiang, Hong Wang, Qianheng Duan, Zhi Ma, and Gui-Lu Long. Fixed-point oblivious quantum amplitude-amplification algorithm. *Scientific Reports*, 12(1):14339, Aug 2022.

[12] Christoph Sünderhauf. Generalized quantum singular value transformation, 2023.

[13] Guang Hao Low and Yuan Su. Quantum eigenvalue processing. In *2024 IEEE 65th Annual Symposium on Foundations of Computer Science (FOCS)*, page 1051–1062. IEEE, October 2024.

[14] Changpeng Shao. Computing eigenvalues of diagonalizable matrices on a quantum computer. *ACM Transactions on Quantum Computing*, 3(4):1–20, 2022.

[15] Changpeng Shao and Jin-Peng Liu. Solving generalized eigenvalue problems by ordinary differential equations on a quantum computer. *Proceedings of the Royal Society A: Mathematical, Physical and Engineering Sciences*, 478(2262), 2022.

[16] George Arfken. *Mathematical Methods for Physicists*. Academic Press, Inc., San Diego, third edition, 1985.

[17] Pedro CS Costa, Dong An, Yuval R Sanders, Yuan Su, Ryan Babbush, and Dominic W Berry. Optimal scaling quantum linear-systems solver via discrete adiabatic theorem. *PRX quantum*, 3(4):040303, 2022.
